# Association of Walnut Consumption with Total and Cause-Specific Mortality and Life Expectancy in U.S. Adults

**DOI:** 10.3390/nu13082699

**Published:** 2021-08-04

**Authors:** Xiaoran Liu, Marta Guasch-Ferré, Deirdre K. Tobias, Yanping Li

**Affiliations:** 1 Department of Nutrition, Harvard T.H. Chan School of Public Health, Boston, MA 02115, USA; Xiaoran_Liu@rush.edu (X.L.); mguasch@hsph.harvard.edu (M.G.-F.); dtobias@bwh.harvard.edu (D.K.T.); 2 Department of Internal Medicine, Rush University Medical Center, Chicago, IL 60612, USA; 3 Channing Division of Network Medicine, Department of Medicine, Brigham and Women’s Hospital and Harvard Medical School, Boston, MA 02115, USA; 4 Division of Preventive Medicine, Department of Medicine, Brigham and Women’s Hospital and Harvard Medical School, Boston, MA 02115, USA

**Keywords:** nuts, walnut, mortality, cardiovascular death, life expectancy

## Abstract

Walnut consumption is associated with health benefits. We aimed to (1) examine the association between walnut consumption and mortality and (2) estimate life expectancy in relation to walnut consumption in U.S. adults. We included 67,014 women of the Nurses’ Health Study (1998–2018) and 26,326 men of the Health Professionals Follow-up Study (1998–2018) who were free of cancer, heart disease, and stroke at baseline. We used Cox regression models to estimate hazard ratios (HRs) and 95% confidence intervals (CIs). During up to 20 years of follow-up, we documented 30,263 deaths. The hazard ratios for total mortality across categories of walnut intake (servings/week), as compared to non-consumers, were 0.95 (95% confidence interval (CI), 0.91, 0.98) for <1 serving/week, 0.94 (95% CI, 0.89, 0.99) for 1 serving/week, 0.87 (95% CI, 0.82, 0.93) for 2–4 servings/week, and 0.86 (95% CI, 0.79, 0.93) for >=5 servings/week (*p* for trend <0.0001). A greater life expectancy at age 60 (1.30 years in women and 1.26 years in men) was observed among those who consumed walnuts more than 5 servings/week compared to *non*-consumers. Higher walnut consumption was associated with a lower risk of total and CVD mortality and a greater gained life expectancy among U.S. elder adults.

## 1. Introduction

In the U.S. diet, intakes of nuts and seeds have increased from 0.5 serving/d to 0.75 serving/d from 1999 to 2012 [1]. The increased consumption of nuts partly contributed to a modest improvement in dietary quality among US adults [1].

Nuts are nutrient-dense foods rich in unsaturated fats, proteins, vitamins, minerals, and fibers [2]. Consumption of nuts is associated with a lower risk of cardiovascular diseases (CVD) [3,4,5], obesity [6,7,8], and type 2 diabetes (T2D) [4] in different populations from varying regions. Walnuts are among the most commonly consumed tree nuts worldwide [9]. Compared to other nuts (i.e., almonds, hazelnuts, pistachio) that are rich sources for monounsaturated fatty acids, walnuts contain high content of alpha-linolenic acid (ALA), a plant-based omega-3 fatty acid [10], which might confer them additional antiatherogenic properties [11] through improving blood lipids [12,13] and endothelial function [14,15].

Walnut consumption has been associated with lower mortality risk [16]. Previous studies that investigated nuts in relation to total mortality and cause-specific mortality [17,18,19] have generally assessed the associations in terms of hazard ratios of the event. These measures of association may limit the translation of results to the general public in an intuitive measure. Therefore, in the present study, we first examine the association between walnut consumption with total mortality and mortality from cardiovascular disease and cancer in two independent, prospective cohorts of U.S. adults. We further estimate the gained life expectancy associated with walnut consumption to convey our findings to support the public health practice.

## 2. Materials and Methods

### 2.1. Study Population

The Nurses’ Health Study (NHS) is a prospective cohort study of 121,701 female nurses aged 30–55 when first enrolled in 1976. The Health Professionals Follow-up Study (HPFS) was established in 1986, with 51,529 male U.S. health professionals (dentists, optometrists, osteopaths, podiatrists, pharmacists, and veterinarians) aged 40–75 years. In both cohorts, follow-up questionnaires are sent every two years to update medical and lifestyle information and identify newly diagnosed cases of various diseases. The self-reported food frequency questionnaires (FFQs) have been completed every four years [10,11]. The study protocol was approved by the institutional review boards of the Brigham and Women’s Hospital and Harvard T.H. Chan School of Public Health, and those of participating registries, as required. 

### 2.2. Dietary Assessment Using FFQ

Dietary intake was assessed by a validated 130 items semi-quantified FFQ administered every 2–4 years. Participants reported how often, on average, they had consumed a particular amount of walnuts, other tree nuts, and peanuts, respectively, during the previous year [15]. For this analysis, we defined 1998 as the baseline year, as this was when questions specific to walnut consumption were first available. We asked participants how often they consumed a serving of walnuts (serving size, 28 g (1 oz)) during the preceding year: never or almost never, less than once per week, once per week, and 2 or more times per week. 

We excluded participants who had cancer, myocardial infarction, and stroke. We excluded participants with (1) implausible energy intake (<600 or >3500 kcal/d for women or <800 or >4200 kcal/d for men) or (2) with missing data reporting walnut consumption baseline and during follow-up. At baseline, 67,014 nurses averagely aged 63.6 years and 26,326 health professionals aged 63.3 years had valid dietary data on walnut consumption. Previous validation study on FFQ demonstrated that nut intake was reported with reasonable accuracy, indicating a correlation coefficient of 0.75 between intake assessed on the baseline dietary questionnaire and intake assessed on four 1 week dietary records. Information on potential confounders, such as age, ethnicity, medical conditions (presence of diabetes, hypertension, or elevated cholesterol), and family history was collected via self-reported biennial questionnaires. Lifestyle factors were collected every 2 years. Diet quality was assessed using the Alternate Healthy Eating Index (AHEI) score, which is based on foods and nutrients predictive of chronic disease risk. Briefly, points were assigned for intake of each component on a scale from 0 to 10, with 10 indicating adherence to the recommended levels of servings per day. We included 10 components of the index in our diet score (walnuts or total nuts were excluded from the calculation): high intakes of vegetables, fruit, nuts, whole grains, polyunsaturated fatty acids, and long-chain omega-3 fatty acids, and low intakes of red and processed meats, sugar sweetened beverages, trans fat, and sodium, as well as moderate alcohol consumption. 

### 2.3. Ascertainment of Total and Cause-Specific Deaths

In NHS and HPFS, deaths were identified from state vital statistics records, the National Death Index, reports by family members, and the postal system in response to the follow-up questionnaires. The follow-up for death in both cohorts was at least 98% complete using these methods [18]. A physician reviewed death certificates or medical records to classify the cause of death using the International Classification of Diseases. The 8th version was used in the NHS and the 9th version in the HPFS.

### 2.4. Statistics

We used Cox proportional hazards models to calculate the adjusted HRs of all-cause and cause-specific mortality risk with their 95% confidence intervals (CIs) across categories of walnut as compared with non-consumers. In the present study, we leverage the repeated measures to construct the model by updating covariates of lifestyle, physical activity, and dietary intakes every 4 years. We used updated levels of dietary intakes and lifestyle factors to examine the association between walnut consumption and mortality. For example, in NHS, we examined the cases of mortality that occurred between 1998 and 2002 in relation to walnut consumption in 1998, and the death cases that occurred between 2002 and 2006 in relation to walnut consumption in 2002, and so forth. The same analytical approach was applied in HPFS. Multivariable model (Model 1) was adjusted for covariates updated over time including age (continuous), sex, race (Caucasian, yes/no), smoking status (never, past, current 1 to 14 cigarettes/day, current 15 to 24 cigarettes/day, current ≥25 cigarettes/day), alcohol consumption (g/day: 0, 1–4.9, 5–14.9, 15–29.9, ≥30), physical activity (metabolic equivalent hours/week, <3, 3–8.9, 9–17.9, 18–26.9, ≥27), current multivitamin use (yes/no), current aspirin use (yes/no), family history of diabetes mellitus (yes/no), myocardial infarction (yes/no) or cancer (yes/no), and menopausal status and hormone use (premenopausal, postmenopausal never users, postmenopausal past users, postmenopausal current users, women only). Model 2 was additionally adjusted for the updated body mass index, history of diabetes mellitus (yes/no), hypertension (yes/no), or hypercholesterolemia (yes/no). In Model 3, we further mutually adjusted with the consumption of other nuts (excluding walnuts). In the final model (Model 4), in the analyses, we accounted for additional consumption of other foods: fruits, vegetables, sugar sweetened beverage, meat, dairy products, whole grain, and refined grains, and total energy intake. Walnut intake was also analyzed as a continuous variable (per 1 serving (28 g) increase) to estimate the HR of mortality per 0.5 inverse in walnut consumption. As sensitivity analyses, we stratified our analysis of walnut and mortality by participants’ background diet quality. A suboptimal diet was defined by an AHEI score less than the median of cohort distribution, whereas an optimal diet quality was an AHEI score above the median. We have evaluated the proportional hazards assumption with a likelihood-ratio test comparing the model with and without an interaction term between time period and walnut consumption. The *p*-value for the proportional hazards assumption was 0.8, indicating that the proportional hazards assumption was not violated in our analyses.

### 2.5. Estimation of Total Life Expectancy

We estimated the life expectancy of U.S. men and women aged 60 years or older, according to the frequency of walnut consumption, using a life table method. The analysis included three parts: (1) age- and sex-specific incident rates (IR) of mortality among participants with different walnut consumption frequencies; (2) the proportion (P) of the population who have consumed walnut with different frequencies; (3) the relative risk (RR) of mortality comparing walnut consumers with non-consumers. 

We inferred the age-specific mortality rates appropriate for our reference group (those who never or almost never consume walnuts).

IRa0 as:

IRa0=IRa(Pa0+∑_(j=1)^4〖Paj×RRaj〗)

where IRa is the population IR of mortality for age group a (of Americans from the CDC WONDER database), Paj is the age-specific proportion of exposure group j, and RRaj is the age-specific RR in comparison of walnut consumption group j versus reference group (j = 0). The age-specific mortality rates in each of the non-reference exposure groups will be then inferred in turn by multiplying the age-specific mortality rate for the reference group IRa0 by the age-specific relative risk: RRaj. 

Finally, a simple life table for each exposure group was built based on each sex- and age-specific IRaj. The gain in life expectancy according to different exposure groups (with different walnut consumption frequencies) was estimated as a difference in the life expectancy at any given age between the reference group (non-consumer) and each group of the walnut consumption frequencies. In a sensitivity analysis, we applied the same method to estimate the association between life expectancy and consumption of total nuts.

## 3. Results

During an average of 17.6 years of follow-up of nurses, we documented 20,655 cases of death, including 3219 cases from CVD and 4496 from cancer among women in NHS (1,178,698 person-year). In HPFS, there were 9608 cases of death consisting of 2663 from CVD and 2210 from cancer in men, with an average of 17.1 years of follow-up (449,861 person-year). Consumption of walnuts increased in these cohorts from an average 0.03 serving/day in 1998 to 0.14 serving/day in 2014. Participants with a higher frequent consumption of walnuts tend to be more physically active, have a healthier diet, lower alcohol consumption, and take multivitamins (Table 1). 

### 3.1. Walnut Consumption and Total Mortality

Age-adjusted and multivariate-adjusted analyses showed that walnut consumption was inversely associated with total mortality in both women and men (Table 2). In comparison with *non*-consumers, the pooled multivariate hazard ratios for total mortality were 0.95 (95% CI: 0.91, 0.98) for those who eat walnuts <1 time per week, 0.94 (95% CI: 0.89, 0.99) for 1 time per week, 0.87 (95% CI: 0.82, 0.93) for 2–4 times per week, and 0.86 (95% CI: 0.79, 0.93) for >5 times per week (*p* for trend < 0.0001). Per 0.5 serving increase in walnut consumption per day was associated with 9% lower risk for total mortality (Hazard Ratio (HR): 0.91, 95%CI: 0.88, 0.95). The inverse dose-response association between walnut consumption and total mortality remained unaffected after we further excluded the *non*-consumers. Per 0.5 serving increase in walnut consumption per day was associated with a hazard ratio of 0.92 (95% CI: 0.89, 0.96) for total mortality among walnut consumers. 

### 3.2. Walnut Consumption and Cause-Specific Mortality

In a pooled analysis of both cohorts, significant inverse associations were observed for deaths due to cardiovascular diseases (Table 2). When comparing extreme quintiles, those who eat walnut >5 times per week had around 25% (HR: 0.75, 95% CI: 0.62, 0.92) lower risk of dying from CVD than *non*-consumers. Per 0.5 serving/day increase in walnut consumption was associated with 14% (HR: 0.86, 95% CI: 0.79, 0.94) lower risk of dying from cardiovascular diseases. Walnut consumption was not associated with cancer mortality in multivariate-adjusted analyses.

**Figure 1:**
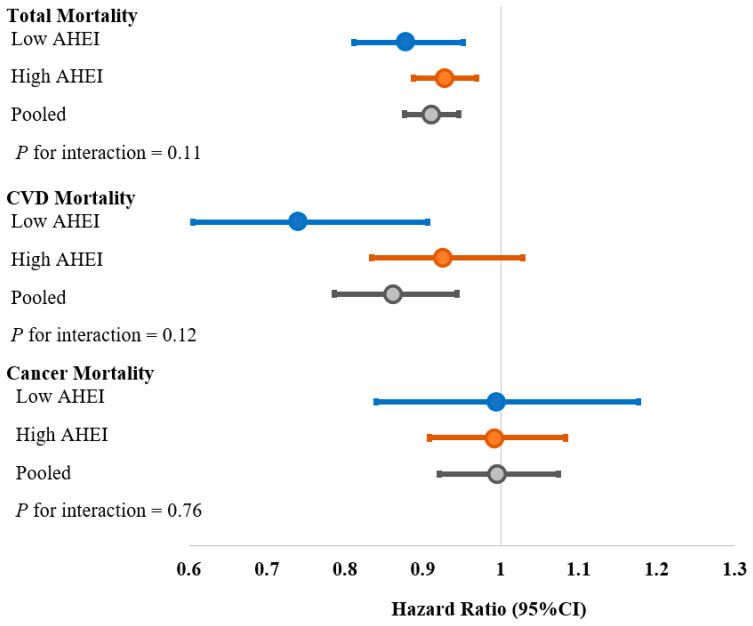
HRs (95% CIs) of total and cause-specific mortality per 0.5 serving increase in walnut consumption stratified by AHEI. Adjusted for covariates updated over time, including: age (continuous), sex, race (Caucasian, yes/no), smoking status (never, past, current 1 to 14 cigarettes/day, current 15 to 24 cigarettes/day, current ≥25 cigarettes/day), alcohol consumption (g/day: 0, 1–4.9, 5–14.9, 15–29.9, ≥30), physical activity (metabolic equivalent hours/week, <3, 3–8.9, 9–17.9, 18–26.9, ≥27), current multivitamin use (yes/no), current aspirin use (yes/no), family history of diabetes mellitus (yes/no), myocardial infarction (yes/no) or cancer (yes/no), and menopausal status and hormone use (premenopausal, postmenopausal never users, postmenopausal past users, postmenopausal current users, among women only), body mass index, history of diabetes mellitus (yes/no), hypertension (yes/no), or hypercholesterolemia (yes/no), other nuts, fruits, vegetables, sugar sweetened beverage, meat, dairy products, whole grain, and refined grain (serving/day), and total energy intake (kcal/day).

We further stratified the analyses by AHEI to examine whether the observed association was related to overall diet quality. The association of walnut consumption and mortality was independent of the background diet of participants (Figure 1). Among participants with a suboptimal diet, 0.5 serving/day increase in walnut consumption was associated with a reduced risk of total mortality (HR: 0.88, 95% CI, 0.81–0.95) and CVD mortality (HR: 0.74, 95% CI, 0.60–0.91) but not cancer mortality (HR: 0.99, 95% CI, 0.84–1.18). Those participants with an optimal diet quality had HR of 0.93 (95% CI, 0.89–0.97), 0.93 (95% CI, 0.83–1.03), and 0.99 (95%CI, 0.91, 1.08) for total, CVD, and cancer mortality, respectively (Figure 1).

### 3.3. Walnut Consumption and Life Expectance

**Figure 2:**
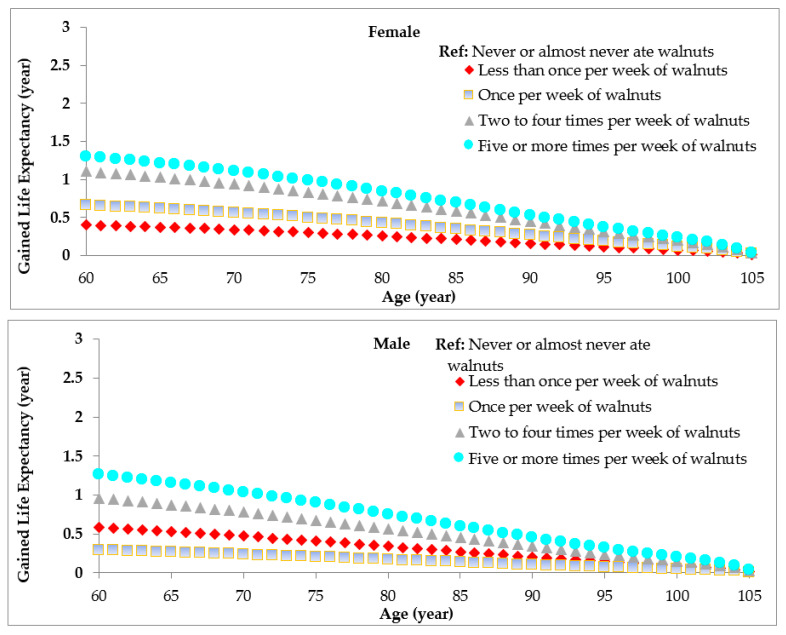
Estimated life expectancy by age and sex according to the frequency of walnut consumption. Overall life expectancy is projected from the overall mortality rate of Americans from Centers of Disease Control and Prevention (CDC) Report.

Using age and sex-specific HRs, we projected that at age 60, women with walnut consumption >5 times per week could potentially gain 1.3 years of life expectancy compared to those who were *non*-consumers, which was 1.26 for men with walnut consumption >5 times per week (Figure 2). That gain of life expectancy at age 60 years is 1.11 years for women and 0.96 years for men who eat walnuts 2–4 times per week (Figure 2). In a sensitivity analysis, we also estimated the association between life expectancy and total consumption of nuts. Compared to *non*-consumers, the potentially prolonged life expectancy at age 60 years was 2.43 years for women and 1.56 years for men among those who consumed nuts >5 times/week (Supplementary Figure S1).

## 4. Discussion

During a follow-up period of up to 20 years, we observed that participants with higher amounts of walnut consumption, as well as the frequency, had a lower risk for all-cause mortality and CVD mortality compared with *non*-consumers. Per 0.5 increase in daily walnut consumption was associated with 9.0% lower risk of total mortality and 14% of CVD mortality, independent from background diet quality and other potential risk factors of participants. There was around one year gained life expectancy at age 60 when compared with the extreme category of walnut consumption.

Our results are consistent with previous evidence supporting the cardiometabolic benefits of nuts. The Adventist Health Study showed that consuming nuts (peanuts, walnuts, almonds) five or more times per week was associated with reduced total mortality [20]. Earlier analysis from the NHS and HPFS all showed significant inverse associations between walnut intake and total mortality [17]. These observations are supported by the results from the PREDIMED (Prevención con Dieta Mediterránea) study, a randomized trial of a Mediterranean diet supplemented with extra virgin olive oil or nuts for the primary prevention of cardiovascular events [21]. Among PREDIMED study participants, when compared to *non*-consumers, individuals who consumed walnuts >3 servings/week had 45% lower risk for total mortality and 47% for CVD mortality during a median follow-up of 4.8 years [16]. Although a similar inverse association with all-cause and CVD mortality was also observed among consumers of ‘other nuts’, the reduced risk of cancer mortality was only associated with consumption of walnuts [16]. In the present analysis, we observed that the inverse association between walnut and cancer mortality remained non-significant after multivariate adjustment. A combined analysis of 31 studies found 15% lower overall cancer risk associated with eating one ounce of nuts per day [22]. Nevertheless, only links between colorectal and endometrial cancers were statistically significant. In a small, short-term intervention study, eating 2 ounces of walnuts per day for 2 weeks could suppress breast cancer cell growth [23]. A secondary analysis of the PREDIMED study demonstrated that the Mediterranean diet with nuts did not decrease the risk for breast cancer compared to the control diet [24]. The relationship of walnuts and cancer risk remains inconsistent and further research is warranted to clarify the effects of walnuts on inflammation, oxidative stress, and cancer risk.

There are several potential mechanisms to which walnut consumption is cardioprotective, including a reduction in risk factors for all-cause mortality such as obesity [8,25], reduction in oxidative stress [26,27], and improvements in endothelial function [15,28] and blood lipids [12]. A plausible explanation for the robustness association observed for walnut is its high content of ALA (a plant omega-3 fatty acid). Indeed, in the Lyon Diet Heart Study, a randomized secondary prevention trial demonstrated that the cardioprotective effects of a Mediterranean diet rich in alpha-linolenic acid were maintained up to 4 years after the first cardiac event [29].

To complement current knowledge, we further translate our findings into more intuitive measures, i.e., life expectancy, to facilitate conveying the results to the general public. We observed that a higher frequency of walnut consumption was associated with a modest gain in life expectancy. These results are in line with previous observations on dietary quality and longevity. A suboptimal dietary quality represents a leading, modifiable cause of morbidity and mortality. Although the overall dietary quality modestly improved among U.S. adults, there is still much potential for improvement. Our findings suggest that incorporating walnuts in diet may potentially contribute to improving overall dietary quality that has been associated with increases in life expectancy among adults in the U.S. [30,31] and other countries [32,33].

Our study has limitations. First, nut intake was self-reported which may introduce measurement error. However, by leveraging the repeated measures of diet every 4 years, we decreased measurement error from within-person variability. Moreover, because dietary data was collected prospectively, errors are likely random with respect to life expectancy, although changes to diet due to diagnoses of chronic diseases or health events cannot be excluded. However, bias due to changes in healthful behaviors motivated by declining health are likely to bias associations of diet towards a higher risk of mortality, not lower. Second, although we adjusted for a number of potential confounders collected repeatedly over follow-up, confounding by unmeasured diet and lifestyle factors correlated with walnut intake is possible. Third, restriction of the study population to health professions could limit the generalizability of the results. However, the homogeneity of the study population could minimize certain residual confounding. Our study has strengths, including the long follow-up of two large cohorts and the repeated measures on detailed diet and lifestyle variables. Another strength is the combination of cohort estimates with the NHANES, a nationally representative cohort, which improved the generalizability of our results. 

## 5. Conclusions

We reported that higher consumption of walnut was associated with a lower risk of all-cause mortality and CVD mortality in two large prospective studies of U.S. elder adults, especially among those with suboptimal dietary quality. We estimated a greater life expectancy at age 60 of 1.3 years in women and 1.26 years in men, among those who consumed walnuts more than 5 servings/week compared to *non*-consumers.

## Figures and Tables

**Table 1:** Baseline characteristics according to frequency of walnut consumption.

	Never or Almost Never	Less Than Once per Week	Once per Week	Two to Four Times per Week	Five or More Times per Week
**Nurses’ Health Study**	(*n* = 51,013)	(*n* = 12,398)	(*n* = 2525)	(*n* = 806)	(*n* = 272)
Age, years	63.8 (7.1)	62.9 (6.7)	63.6 (6.9)	65.0 (6.8)	64.8 (6.7)
BMI	26.7 (5.3)	26.5 (5.2)	26.2 (4.9)	25.9 (5.2)	25.0 (5.1)
AHEI score	46.0 (9.1)	47.7 (8.8)	49.8 (8.9)	53.5 (9.7)	57.4 (9.6)
Physical activity, MET h/wk	16.9 (21.3)	19.1 (21.6)	20.5 (22.9)	22.7 (25.5)	23.1 (24.4)
Alcohol intake, g	5.0 (9.2)	5.1 (8.8)	5.1 (8.2)	4.4 (7.8)	4.7 (9.1)
Smoking status					
Past smoker,%	45.0	43.0	43.4	38.8	46.8
Current smoker, %	11.2	8.3	6.6	6.7	2.6
White, %	97.5	97.9	98.0	95.4	97.7
Multivitamin use, %	60.4	62.3	63.7	67.2	74.6
Aspirin use, %	50.2	52.8	53.1	51.2	50.4
Family history of diabetes, %	28.5	28.4	29.1	27.8	31.4
Family history of cancer, %	12.8	12.8	14.0	11.0	13.7
Family history of myocardial infarction, %	24.9	24.7	23.5	23.1	23.2
Family history of Hypercholesterolemia, %	34.1	33.3	34.9	34.3	36.7
Family history of Diabetes, %	6.8	5.7	5.4	5.5	8.0
Family history of Hypertension, %	31.6	30.4	27.6	27.0	23.0
**Health Professionals** **Follow-Up Study**	(*n* = 20,000)	(*n* = 4657)	(*n* = 1068)	(*n* = 387)	(*n* = 214)
Age, years	63.2 (8.9)	63.6 (8.8)	64.0 (8.5)	65.0 (8.9)	65.7 (8.4)
BMI	26.1 (3.6)	26.1 (3.6)	25.8 (3.3)	25.3 (3.2)	25.4 (3.2)
AHEI score	45.1 (9.7)	47.2 (9.3)	48.9 (9.4)	53.5 (10)	56.4 (10.4)
Physical activity	31.4 (30.3)	36.2 (33.1)	36.6 (33.8)	39.5 (33.7)	47.4 (43.1)
Alcohol intake, g	11.2 (14.3)	10.9 (13.2)	10.9 (14.8)	9.7 (12.6)	9.7 (12.1)
Smoking status					
Past smoker,%	51.1	48.4	47.3	46.4	47.0
Current smoker, %	4.9	3.8	4.0	5.0	4.1
White, %	91.7	92.1	92.4	90.9	89.6
Multivitamin use, %	48.2	50.2	52.6	55.1	54.3
Aspirin use, %	65.0	66.0	63.8	64.3	60.3
Family history of diabetes, %	22.6	21.9	22.8	20.9	24.6
Family history of cancer, %	37.5	38.8	39.2	37.5	33.8
Family history of myocardial infarction, %	32.4	30.9	31.1	30.7	29.3
Family history of hypercholesterolemia, %	28.7	27.0	27.5	30.5	27.5
Family history of diabetes, %	6.0	5.3	6.8	6.1	4.5
Family history of hypertension, %	26.6	24.6	24.7	25.4	22.9

Values are means (SD). All variables except age are age-standardized. Percentages for categorical variables are standardized to the age distribution of the study population.

**Table 2:** Total mortality, specific cause of mortality according to frequency of walnut consumption.

	Never or Almost Never	<1 per Week	1 per Week	2–4 Times per Week	≥5 Times per Week	Per 0.5 Serving Increase	*p* for Trend
**Total mortality**							
Women							
No. of person-years	763,683	245,165	82,129	55,752	31,969		
No. of deaths	15,469	3110	966	701	409		
Age-adjusted model	1.00	0.64 (0.62,0.67)	0.57 (0.53,0.60)	0.49 (0.45,0.53)	0.47 (0.42,0.52)	0.56 (0.53,0.59)	<0.0001
Multivariate-adjusted Model 1	1.00	0.92 (0.89,0.96)	0.87 (0.81,0.92)	0.80 (0.74,0.86)	0.79 (0.71,0.87)	0.85 (0.81,0.89)	<0.0001
Multivariate-adjusted Model 2	1.00	0.94 (0.90,0.98)	0.87 (0.82,0.93)	0.80 (0.74,0.87)	0.78 (0.71,0.86)	0.85 (0.81,0.89)	<0.0001
Multivariate-adjusted Model 3	1.00	0.96 (0.92,1.00)	0.93 (0.87,0.99)	0.87 (0.80,0.94)	0.85 (0.77,0.94)	0.90 (0.86,0.95)	<0.0001
Multivariate-adjusted Model 4	1.00	0.95 (0.91,0.99)	0.92 (0.86,0.99)	0.87 (0.81,0.95)	0.85 (0.77,0.94)	0.90 (0.86,0.95)	<0.0001
Men							
No. of person-years	292,832	90,907	31,655	19,193	15,274		
No. of deaths	6809	1605	573	347	274		
Age-adjusted model	1.00	0.75 (0.71,0.79)	0.73 (0.67,0.79)	0.62 (0.55,0.69)	0.59 (0.52,0.66)	0.71 (0.67,0.75)	<0.0001
Multivariate-adjusted Model 1	1.00	0.93 (0.88,0.98)	0.94 (0.86,1.03)	0.87 (0.78,0.96)	0.82 (0.73,0.93)	0.89 (0.84,0.95)	0.0001
Multivariate-adjusted Model 2	1.00	0.94 (0.89,0.99)	0.95 (0.87,1.03)	0.86 (0.77,0.96)	0.82 (0.72,0.92)	0.89 (0.84,0.94)	<0.0001
Multivariate-adjusted Model 3	1.00	0.95 (0.89,1.00)	0.98 (0.89,1.07)	0.90 (0.80,1.01)	0.86 (0.76,0.98)	0.92 (0.87,0.98)	0.007
Multivariate-adjusted Model 4	1.00	0.93 (0.88,0.99)	0.97 (0.88,1.06)	0.89 (0.80,1.00)	0.86 (0.76,0.98)	0.92 (0.87,0.98)	0.008
Pooled							
Age-adjusted model	1.00	0.68 (0.65,0.70)	0.62 (0.59,0.65)	0.53 (0.49,0.56)	0.51 (0.47,0.55)	0.61 (0.59,0.64)	<0.0001
Multivariate-adjusted Model 1	1.00	0.93 (0.90,0.95)	0.89 (0.85,0.94)	0.82 (0.77,0.87)	0.80 (0.74,0.86)	0.87 (0.83,0.90)	<0.0001
Multivariate-adjusted Model 2	1.00	0.94 (0.91,0.97)	0.90 (0.85,0.94)	0.82 (0.77,0.87)	0.79 (0.74,0.86)	0.86 (0.83,0.89)	<0.0001
Multivariate-adjusted Model 3	1.00	0.96 (0.92,0.99)	0.94 (0.89,0.99)	0.87 (0.81,0.93)	0.85 (0.79,0.92)	0.91 (0.87,0.94)	<0.0001
Multivariate-adjusted Model 4	1.00	0.95 (0.91,0.98)	0.94 (0.89,0.99)	0.87 (0.82,0.93)	0.86 (0.79,0.93)	0.91 (0.88,0.95)	<0.0001
**CVD mortality**							
Women							
No. of CVD deaths	2468	475	139	96	41		
Age-adjusted model	1.00	0.72 (0.65,0.79)	0.60 (0.51,0.72)	0.51 (0.42,0.63)	0.39 (0.29,0.54)	0.52 (0.45,0.60)	<0.0001
Multivariate-adjusted Model 1	1.00	0.97 (0.88,1.07)	0.87 (0.73,1.03)	0.80 (0.65,0.99)	0.62 (0.45,0.84)	0.78 (0.68,0.89)	0.0002
Multivariate-adjusted Model 2	1.00	0.99 (0.89,1.09)	0.87 (0.73,1.04)	0.82 (0.67,1.01)	0.63 (0.46,0.86)	0.79 (0.69,0.90)	0.0004
Multivariate-adjusted Model 3	1.00	1.01 (0.91,1.12)	0.93 (0.78,1.11)	0.89 (0.72,1.10)	0.69 (0.50,0.95)	0.84 (0.73,0.96)	0.02
Multivariate-adjusted Model 4	1.00	1.00 (0.89,1.11)	0.92 (0.77,1.11)	0.90 (0.72,1.11)	0.69 (0.50,0.95)	0.84 (0.73,0.96)	0.01
Men							
No. of CVD deaths	1915	437	162	83	66		
Age-adjusted model	1.00	0.76 (0.69,0.85)	0.79 (0.67,0.93)	0.57 (0.46,0.71)	0.55 (0.43,0.70)	0.67 (0.60,0.76)	<0.0001
Multivariate-adjusted Model 1	1.00	0.93 (0.83,1.03)	1.01 (0.85,1.18)	0.79 (0.63,0.99)	0.73 (0.57,0.93)	0.84 (0.75,0.94)	0.003
Multivariate-adjusted Model 2	1.00	0.95 (0.85,1.05)	1.02 (0.87,1.20)	0.79 (0.63,0.99)	0.73 (0.57,0.94)	0.84 (0.75,0.94)	0.003
Multivariate-adjusted Model 3	1.00	0.94 (0.84,1.05)	1.05 (0.89,1.25)	0.85 (0.68,1.07)	0.82 (0.63,1.06)	0.90 (0.80,1.01)	0.07
Multivariate-adjusted Model 4	1.00	0.93 (0.83,1.04)	1.03 (0.87,1.22)	0.83 (0.66,1.05)	0.80 (0.62,1.03)	0.88 (0.78,0.99)	0.04
Pooled							
Age-adjusted model	1.00	0.74 (0.69,0.79)	0.69 (0.61,0.78)	0.54 (0.46,0.62)	0.48 (0.39,0.58)	0.60 (0.55,0.66)	<0.0001
Multivariate-adjusted Model 1	1.00	0.95 (0.88,1.02)	0.94 (0.84,1.06)	0.80 (0.69,0.93)	0.68 (0.56,0.83)	0.81 (0.74,0.89)	<0.0001
Multivariate-adjusted Model 2	1.00	0.97 (0.90,1.04)	0.95 (0.84,1.07)	0.80 (0.69,0.93)	0.69 (0.57,0.84)	0.82 (0.75,0.89)	<0.0001
Multivariate-adjusted Model 3	1.00	0.98 (0.90,1.05)	0.99 (0.87,1.12)	0.86 (0.74,1.01)	0.76 (0.62,0.93)	0.87 (0.79,0.95)	0.002
Multivariate-adjusted Model 4	1.00	0.97 (0.89,1.04)	0.98 (0.86,1.11)	0.86 (0.73,1.00)	0.75 (0.62,0.92)	0.86 (0.79,0.94)	0.001
**Cancer mortality**							
Women							
No. of cancer deaths	3225	768	254	154	95		
Age-adjusted model	1.00	0.83 (0.77,0.90)	0.79 (0.69,0.89)	0.65 (0.55,0.77)	0.74 (0.60,0.91)	0.77 (0.70,0.85)	<0.0001
Multivariate-adjusted Model 1	1.00	1.05 (0.96,1.13)	1.05 (0.92,1.20)	0.92 (0.78,1.08)	1.06 (0.86,1.30)	1.01 (0.92,1.10)	0.91
Multivariate-adjusted Model 2	1.00	1.05 (0.97,1.14)	1.06 (0.93,1.20)	0.92 (0.78,1.09)	1.05 (0.85,1.29)	1.00 (0.91,1.10)	0.95
Multivariate-adjusted Model 3	1.00	1.04 (0.95,1.13)	1.07 (0.93,1.22)	0.94 (0.79,1.11)	1.09 (0.88,1.35)	1.03 (0.93,1.13)	0.60
Multivariate-adjusted Model 4	1.00	1.02 (0.94,1.12)	1.06 (0.92,1.21)	0.94 (0.80,1.12)	1.10 (0.89,1.36)	1.03 (0.93,1.13)	0.61
Men							
No. of cancer deaths	1476	431	151	86	66		
Age-adjusted model	1.00	0.93 (0.84,1.04)	0.90 (0.76,1.07)	0.77 (0.62,0.96)	0.72 (0.56,0.92)	0.82 (0.73,0.92)	0.0009
Multivariate-adjusted Model 1	1.00	1.07 (0.96,1.19)	1.07 (0.90,1.27)	0.97 (0.78,1.21)	0.93 (0.73,1.19)	0.97 (0.86,1.08)	0.57
Multivariate-adjusted Model 2	1.00	1.08 (0.97,1.20)	1.07 (0.91,1.27)	0.96 (0.77,1.19)	0.91 (0.71,1.17)	0.96 (0.85,1.07)	0.43
Multivariate-adjusted Model 3	1.00	1.06 (0.95,1.19)	1.04 (0.87,1.24)	0.92 (0.73,1.15)	0.87 (0.67,1.13)	0.93 (0.82,1.05)	0.22
Multivariate-adjusted Model 4	1.00	1.07 (0.95,1.20)	1.05 (0.88,1.25)	0.93 (0.74,1.17)	0.91 (0.70,1.18)	0.95 (0.84,1.07)	0.37
Pooled							
Age-adjusted model	1.00	0.86 (0.81,0.92)	0.82 (0.74,0.91)	0.69 (0.60,0.78)	0.73 (0.62,0.85)	0.79 (0.73,0.85)	<0.0001
Multivariate-adjusted Model 1	1.00	1.06 (0.99,1.13)	1.06 (0.96,1.18)	0.94 (0.82,1.07)	1.01 (0.86,1.18)	0.99 (0.92,1.07)	0.85
Multivariate-adjusted Model 2	1.00	1.06 (0.99,1.13)	1.06 (0.96,1.18)	0.94 (0.82,1.07)	1.00 (0.85,1.17)	0.99 (0.92,1.06)	0.76
Multivariate-adjusted Model 3	1.00	1.05 (0.98,1.12)	1.06 (0.95,1.18)	0.93 (0.81,1.07)	1.00 (0.85,1.18)	0.99 (0.92,1.06)	0.73
Multivariate-adjusted Model 4	1.00	1.04 (0.97,1.12)	1.05 (0.94,1.17)	0.94 (0.82,1.08)	1.02 (0.86,1.20)	1.00 (0.92,1.07)	0.89

Multivariate adjusted **Model 1** was adjusted for covariates updated over time, including age (continuous), sex, race (Caucasian, yes/no), smoking status (never, past, current 1 to 14 cigarettes/day, current 15 to 24 cigarettes/day, current ≥25 cigarettes/day), alcohol consumption (g/day: 0, 1–4.9, 5–14.9, 15–29.9, ≥30), physical activity (metabolic equivalent hours/week, <3, 3–8.9, 9–17.9, 18–26.9, ≥27), current multivitamin use (yes/no), current aspirin use (yes/no), family history of diabetes mellitus (yes/no), myocardial infarction (yes/no) or cancer (yes/no), and menopausal status and hormone use (premenopausal, postmenopausal never users, postmenopausal past users, postmenopausal current users, among women only). **Model 2** was Model 1 further adjusted for time-varying body mass index, presence of diabetes mellitus (yes/no), hypertension (yes/no), or hypercholesterolemia (yes/no). **Model 3** was Model 2 further mutually adjusted with consumption of other nuts (serving/day). **Model 4** (final model) was model 3 additionally adjusted for consumption of other foods: fruits, vegetables, sugar sweetened beverage, meat, dairy products, whole grain, and refined grain (serving/day), and total energy intake (kcal/day). Frequency of nut consumption pertains to one serving of nuts, defined as 28 g.

## Data Availability

The Health Professionals Follow-up Study, the Nurses’ Health Study, and the Nurses’ Health Study 2 data may be used in collaboration with a principal investigator. Please see the study websites for more information: https://www.hsph.harvard.edu/hpfs/for-collaborators/, and http://www.nurseshealthstudy.org/researchers.

## Supplementary Materials

The following are available online at https://www.mdpi.com/article/10.3390/nu13082699/s1, Figure S1: Flowchart of participant included in the study.
