# Supplementary figures and images for: Association of Walnut Consumption with Total and Cause-Specific Mortality and Life Expectancy in U.S. Adults
Source: Nutrients. 2021 Aug 4;13(8):2699. doi: 10.3390/nu13082699 (PMC8401409; doi:10.3390/nu13082699)

## Supplementary Material

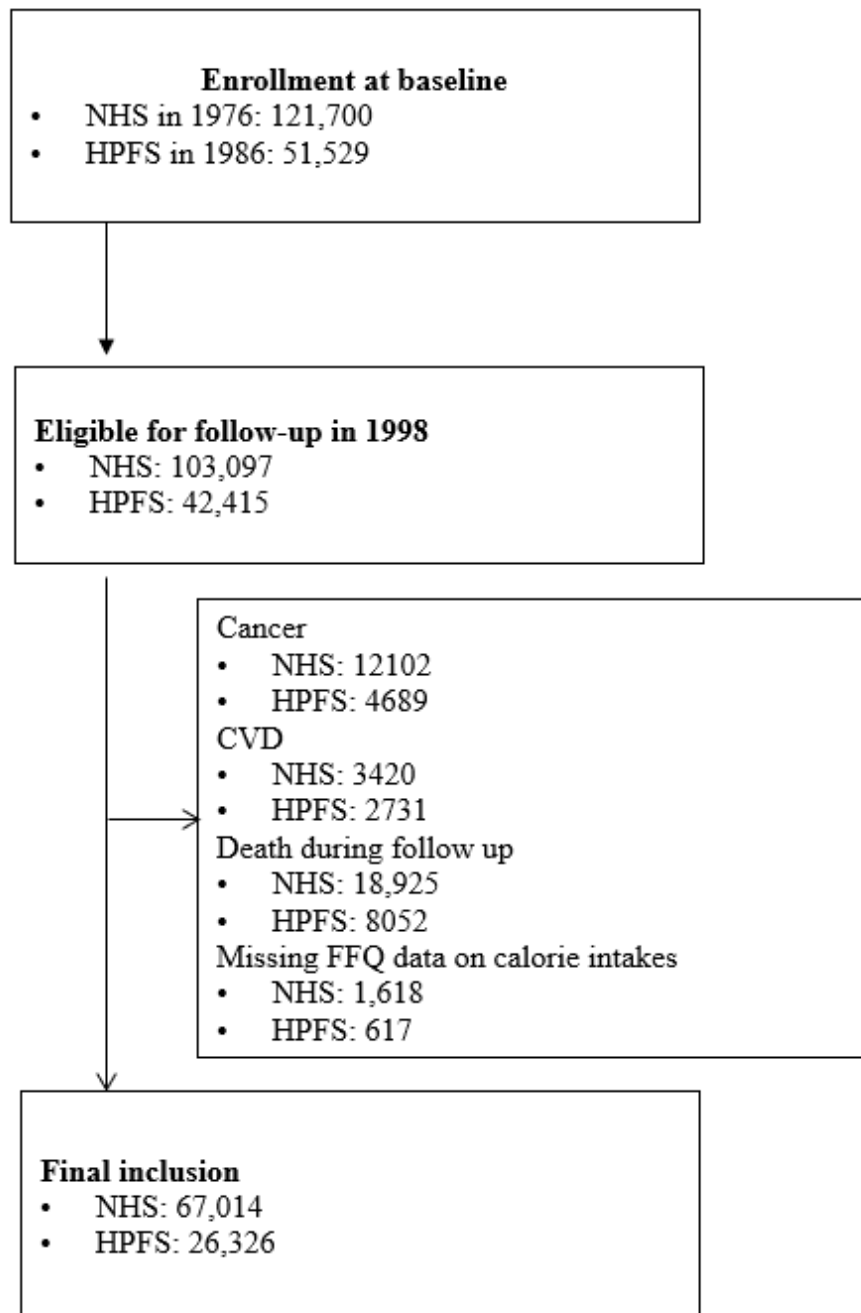

**Figure S1.** Flowchart of participant included in the study.

Supplement: Supplementary file 1 [file nutrients-13-02699-s001.zip › nutrients-1281440-supplementary.pdf]
